# Risk of ozone exposure-induced fracture

**DOI:** 10.3389/fpubh.2023.1153256

**Published:** 2023-03-16

**Authors:** Shuai Lu, Rongrong Xu, Maoqi Gong, Yejun Zha, Ning Li, Jia Chen, Xuejiao Liu, Xieyuan Jiang

**Affiliations:** ^1^Department of Orthopedic Trauma, Beijing Jishuitan Hospital, Beijing, China; ^2^Center for Global Health, School of Public Health, Nanjing Medical University, Nanjing, China; ^3^Department of Endocrinology, Beijing Jishuitan Hospital, Beijing, China; ^4^Department of Medical Record Management and Statistics, Beijing Jishuitan Hospital, Beijing, China

**Keywords:** ozone, fracture, exposure, risk, health effect

## Abstract

**Introduction:**

Ozone (O_3_) is known to induce oxidative stress that influences various cells and tissues, which may further lead to diminished bone mineral density. Nevertheless, few studies have investigated the association between O_3_ exposure and fractures. Considering the similar growing trends of O_3_ concentrations and fracture morbidity in recent years, in the present study, we aimed to examine whether O_3_ exposure is associated with the fracture morbidity.

**Methods:**

Using a retrospective cohort study design, we analyzed the records of 8,075 patients with fracture admitted in the warm season to Beijing Jishuitan Hospital from 2014 to 2019 and matched them to the corresponding exposure time and concentration of O_3_.

**Results:**

The results showed that increased odds of fracture were associated with increased O_3_ concentrations, presumably because O_3_ induces oxidative stress (OS) that leads to bone mineral density (BMD) loss.

**Discussion:**

Our findings suggest that O_3_ exposure is a risk factor for fractures, providing new evidence of the adverse health effect induced by air pollution. We can conclude that more intensive air pollution control is needed for the prevention of fracture occurrence.

## Introduction

Fracture has become a global public health issue causing a serious economic burden (1–3). The global number of new fracture cases was estimated to be 178 million in 2019, showing an increase of 33.4% compared with 1990 (3). The prevalence of fractures is also expected to increase in the coming decades. Apart from the health burdens, the treatment of fractures have also created heavy economic burdens for individuals and healthcare systems (4, 5). For example, the pooled cost for treating hip fractures was estimated to be $10,075, and the global care costs for one hip fracture were $43,669.7 (6). Hence, there is an urgent need to develop effective fracture prevention methods.

Understanding the risk factors of fracture is the key to developing effective preventive measures. To date, epidemiological studies have identified a series of risk factors for fracture, including family history (7), genetic susceptibility (8), physical activity (9), and environmental factors (10). Researchers have also explored the potential effects of air pollutants on bone health. For instance, a meta-analysis showed a particulate matter with diameters <2.5 μm (PM_2.5_) and nitrogen dioxide (NO_2_) are associated with an elevated risk of osteoporosis (11). However, the effect of other air pollutants on bone fracture remains unknown.

Osteoporosis is the most prevalent metabolic bone disease marked by low bone mass, the primary risk factor for fractures (12). Oxidative stress (OS) plays an important role in the development and progression of osteoporosis (13). For example, OS can induce osteoblast dysfunction by upregulating Bach1, which induces apoptosis and further leads to bone mineral density (BMD) loss and osteoporosis (13). Reactive oxygen species (ROS) generation and accumulation is the primary source of OS, which may be induced by the intake of ozone (O_3_), the strongest oxidizing agent of all air pollutants. Therefore, we hypothesized that the inhalation of O_3_ can elevate the risk of osteoporosis, hence increasing the occurrence of fractures.

To validate our hypothesis, in the present study, we collected data from the clinical records of 8,075 patients with fractures obtained in the warm season and treated at Beijing Jishuitan Hospital from 2014 to 2019. We then computed the patients' air pollutant exposure levels by taking the data from the nearest air monitoring stations from their homes. We investigated the associations between the risk of fracture and O_3_ exposure level, which may facilitate the promotion of health care and reduce the risk of fracture.

## Materials and methods

### Study population

The retrospective cohort design was used in the present study. Clinical information was collected from Jishuitan Hospital, Beijing from patients admitted between 2014 and 2019. We recorded the addresses of all subjects. Ethical approval was obtained from Beijing Jishuitan Hospital Ethical Review Committee (No. 202009-16). Since only anonymous clinical information was collected, no individual informed consent was obtained.

### Air pollution exposure assessment

We collected data of the hourly concentrations of six air pollutants for the period 2014–2019 from national air quality monitoring stations. The daily average concentrations of particulate matter with diameters <10 μm (PM_10_), PM_2.5_, carbon monoxide (CO), sulfur dioxide (SO_2_), and NO_2_. Additionally, the daily maximum 8-h average concentrations for O_3_ were calculated from daily hourly data, which was taken as the exposure concentration.

Considering the delayed effects of air pollutants, we set up lag models to assess the effect of O_3_ exposure on the occurrence of fracture. The single-day lag model included 1 (lag 1), 2 (lag 2), 3 (lag 3), 4 (lag 4), 5 (lag 5), 6 (lag 6), and 7 days (lag 7) prior to the day of fracture onset.

### Statistical analysis

Spearman correlation coefficients were calculated to quantify the correlations between air pollutants and fracture. Time-stratified case-crossover design was employed to examine the effect of O_3_ on fracture. Briefly, for each patient, the level of O_3_ exposure before the day of fracture onset was compared with the control periods of the same individual when the event had not occurred (14). This design enabled each individual to be his or her own control and minimized the possible confounding impact factors of other patients, such as demographic, socioeconomic, and behavioral risk factors. For each patient, we created 3 to 4 control index days matched to the case index day by the same day and the day of the week, month, and year. We used conditional logistic regression models to explore the associations between O_3_ exposure and fracture, and assumed that there are linear relationships between them. A distributed lag model was applied to assess the non-linear delayed effect of O_3_ by constructing the cross-basis function, i.e., bi-dimensional functions that simultaneously describe the shape of the relationship along the predictor space and its distributed lag effects (15), which was added to the primary conditional logistic regression model. A maximum of a 7-day lag (current day and 6 days before event onset) was selected, and the lag structure was modeled using quadratic polynomial function. The construction of model was shown in the below:


$$\begin{array}{l} logit\left(P\left(case=1\;in\;stratum\;i\right)\right)\\ \;=a_{stratum\;i}+\beta PDLM\left(ozone\right)+\gamma ns\left(Temp,\;3\right)+\delta ns\left(RH,3\right) \end{array}$$

In the equation, *logit*(*P*(*case* = 1 *in stratum i*)) was the conditional probability of being case in the *i*th stratum; *PDLM*(*ozone*) is a matrix produced by a cross-basis function for ozone concentrations modeled by *PDLM* with a prior second-degree polynomial function for the lag structure and linear function for concentration–response relationships; *ns*(*Temp*, 3) is the natural cubic spline function of 0–24 averaged temperature with 3 degrees of freedom (df); *ns*(*RH*, 3) is the natural cubic spline function of 0–24 averaged relative humidity with 3 df. Because O_3_ concentrations were very low in the cold season, the associations between O_3_ and fracture were evaluated only for the warm season (from May to October). Due to temperature effects on O_3_ formation, we used a three cubic natural spline function to control the potential effect of temperature.

To explore potential effect modifications, stratified analyses were performed by age (≥60 vs. <60 years) and gender (male vs. female). We used the two-sample *t-*test to detect any differences in the stratum-specific estimates. All analyses were performed using the packages “season,” “dlnm,” and “splines” in in the R software version 4.2.2. *P*-values < 0.05 were considered to indicate statistical significance. To facilitate outcome interpretations, we determined the odds ratios and 95% confidence intervals (95% CIs) associated with an interquartile range increase in O_3_ concentrations.

## Results

### Descriptive data

The mean age of the 8,075 fracture cases was 50.86 (18.2) years, and the males accounted for 49.4% of the fracture cases (Table 1). The median of fracture onset was seven cases per day. Table 2 displays the distribution of air pollutants and temperature. The average O_3_ concentration was 85.59 (40.72) μg/m^3^ during the study period, and the mean temperature was 22.89°C. O_3_ was highly correlated with temperature (*r* = 0.7); however, weak correlations with other air pollutants were also observed (Supplementary Figure S1).

**Table 1:** Baseline characteristics of the study population.

**Variable**	**Mean**	**SD**	**Median**	***Q*_1_**	***Q*_3_**	**IQR**
SO_2_	5.58	6.07	3.55	2.66	6.74	4.08
CO	0.86	0.40	0.81	0.58	1.07	0.49
O_3_	82.59	40.73	81.15	50.21	112.76	62.55
NO_2_	44.62	18.84	40.47	32.22	52.95	20.73
PM_10_	82.01	62.01	69.62	44.10	106.14	62.04
PM_2.5_	57.80	45.39	48.11	25.49	75.65	50.17
Temperature	22.89	5.53	24.25	20.00	27.00	7.00
Fracture cases	7.31	4.88	7	3	11	8

SD, standard deviation.

**Table 2:** Summarized statistical data of air pollutants and temperature.

**Baseline characteristics**	
**Age: years**, ***N*** **(%)**	
< 60	5,352 (66.3%)
≥60	2,723 (33.7%)
Age: years; mean (SD)	50.86 (18.2)
**Gender:** ***N*** **(%)**	
Male	3,989 (49.4%)
Female	4,086 (50.6%)

SD, standard deviation; Q_1_, lower quartile; Q_3_, upper quartile; IQR, interquartile range.

### Regression analysis results

**Figure 1:**
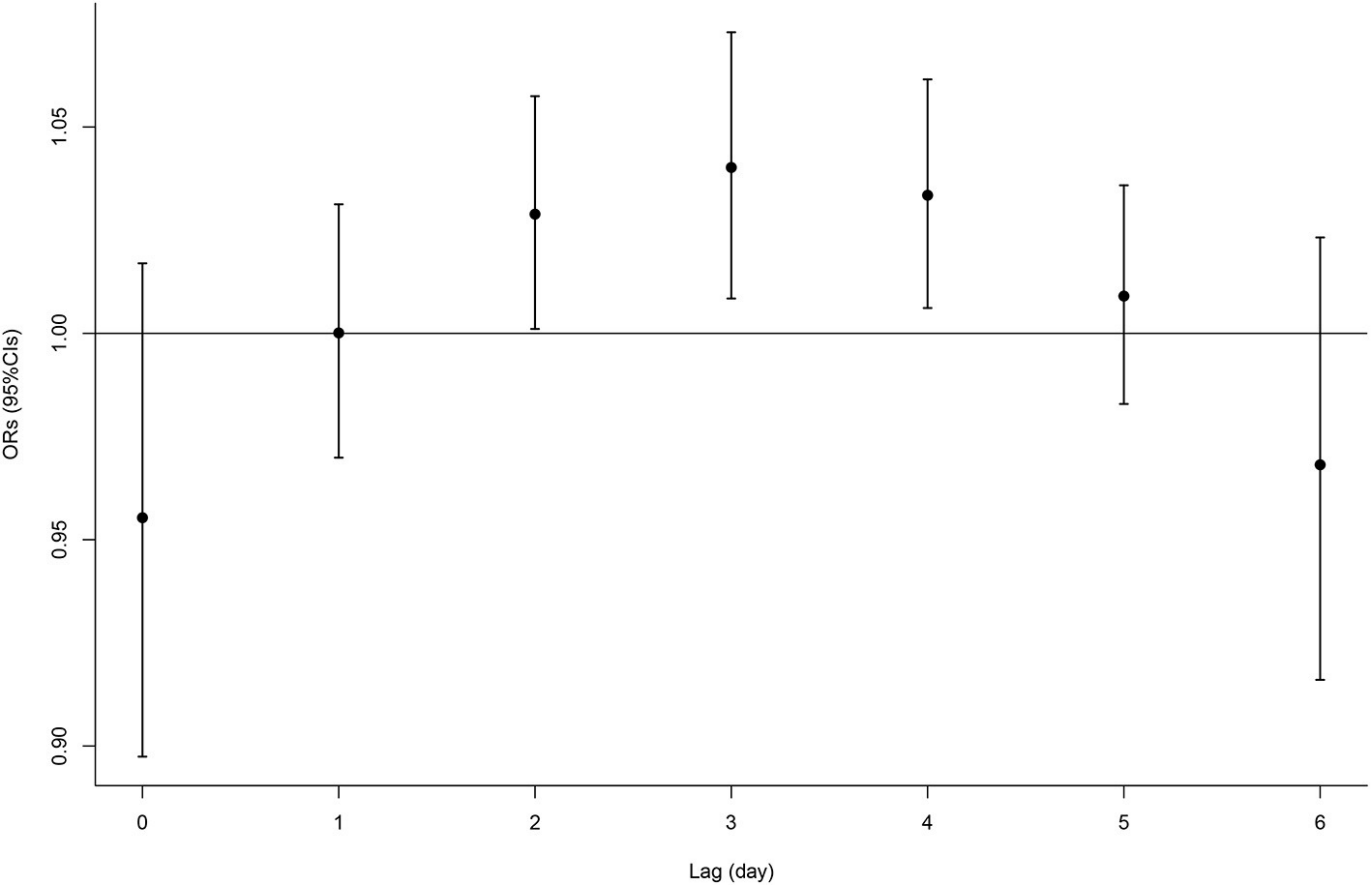
The OR value in occurrence of fracture associated with O_3_ at different lag periods.

The single-lag effect of O_3_ can be seen in the data displayed in Figure 1 and Supplementary Table S1. We observed that the O_3_ exposure from lag 2 to 4 was positively associated with fracture. Then, the associations were diminished and became statistically nonsignificant after lag 5 day. For example, each IQR (63 μg/m^3^) increase in the concentrations of O_3_ at lag 3 was associated with a higher risk of fracture onset by 4% (95% CI, 1–7%). Due to the significant association of O_3_ with fracture from lags 2–4, we calculated the cumulative effect for that period as well as other cumulative effects of the current day. Table 3 presents the results of the cumulative lag effects of O_3_ at different lag periods. We found a cumulative effect of O_3_ only from lag 2–4 days, with no other lag periods. Each IQR increase in O_3_ within lags 2–4 was associated with an OR of 1.11 (95% CI, 1.02–1.20) for fracture.

**Table 3:** ORs and 95% CIs of fracture associated with an IQR increase in ozone over multiple lag days.

**Lag**	**ORs**	**95% CIs**
Lag 0–2	0.98	(0.89, 1.09)
Lag 0–4	1.06	(0.95, 1.18)
Lag 2–4	1.11	(1.02, 1.20)
Lag 0–6	1.03	(0.90, 1.19)

ORs, odds ratios; CIs, confidence intervals.

In our stratified analyses, we observed that the associations between O_3_ and fracture in the female group were still significant, similar to those in the overall population. The single-lag ORs of each IQR increase in O_3_ on males ranged from 0.94 (95% CI, 0.87–1.02) to 1.06 (95% CI, 1.01–1.10). However, there were no significant associations between O_3_ and fracture in males (Supplementary Figure S2). In the stratified analysis in the subgroup of age, we found significant associations between O_3_ exposure from lag 2 to 4 and fracture in the subgroup of age lower than 60 (Supplementary Figure S3). However, the two-sample *t-*test did not detect between-stratum differences in the subgroups (Supplementary Tables S2, S3).

## Discussion

On the basis of extensive clinical evidence of fracture and matching with the air pollution concentrations, the results of this study revealed an association between a higher risk of fracture and the length of O_3_ exposure. Of all the air pollutants we analyzed, we found that fracture was associated only with the concentration of O_3_. Our findings may have important implications in formulating intervention measures to reduce the risk of fracture caused by O_3_ exposure.

Osteoporosis is the most important risk factor for fractures. Previous studies have explored the relationship between air pollution and osteoporosis. Evidence suggests that long-term exposure to PM_2.5_ is associated with a decreased bone mineral density (BMD) and an increased risk of osteoporosis (16) and osteoporotic fracture (17). Long-term exposure to PM_10_ was linked to newly diagnosed osteoporosis in Korean adults (18). Alver et al. (19) also reported that PM_2.5_ and PM_10_ levels were negatively associated with distal forearm BMD. A meta-analysis also confirmed the association between exposure to PM_2.5_ and an elevated risk of osteoporosis (11). Of the other air pollutants, the available evidence also suggested an association between the increased risk of osteoporosis and elevated NO_2_ exposure (20). Moreover, biomass exposure was also associated with an increased risk of bone resorption and consequent osteoporosis (21). Given the above findings, we speculated that air pollutants could lead to the development of osteoporotic changes, which increase the risk of fracture.

Many studies have revealed that OS affects BMD. Evidence has been reported that OS is a risk factor for osteoporosis and that the oxidant/ antioxidant imbalance affects osteoblastic activity (22–25). Moreover, OS is involved in the bone metabolic pathway. The upregulation of the receptor activator of NF-kB ligand (RANKL) and the downregulation of osteoprotegerin (OPG) are associated with the pathogenesis of osteoclastogenesis induced by OS. RANKL acts as the osteoclastic activity activator, whereas OPG is the osteoclastic activity inhibitor, both critical in bone metabolism (26–28). Isolated antioxidants have been proposed to counteract bone loss by reducing the expression of RANKL and increasing the expression of OPG. Animal studies also revealed that antioxidants could increase bone density by mitigating the RANKL activation of osteoclastic activity (29, 30). Moreover, increased osteoblast and osteocyte apoptosis could induce OS. Osteocyte death decreases the levels of cytokines involved in osteoblastic activity, which leads to osteoclastogenesis. Furthermore, dead and dying osteocytes were also found to stimulate further osteoclastogenesis (31–33). These effects are mitigated by antioxidants, such as GSH, alpha-lipoic acid, and N-acetylcysteine (26, 34). Therefore, we conclude that the pathways through which OS exerts effects on BMD are the following. Firstly, OS can increase osteoclastogenesis and osteoblast and osteocyte apoptosis, while decreasing osteoblast activity and osteoprogenitor differentiation in the osteoblast cell lineage. Moreover, PM_2.5_ and NO_2_ exposure induce oxidative stress (OS). O_3_ is also an important source that stimulates the production of endogenous ROS, a common source of OS. Previous studies reported that PM_2.5_ and NO_2_ exposures were associated with an elevated risk of fracture (20). We also found that O_3_ exposure elevated the odds of fracture. Therefore, based on this previous evidence, we speculated that O_3_ exposure-induced OS could be the pathogenesis factor leading to fracture.

This study has several limitations. First, the exposure level was assessed by matching the longitude and latitude of the home address information to the national air quality monitoring stations, but there were some interferences from the pollutants at different microenvironments. Secondly, due to the limited availability of information caused by the retrospective design of the study, some confounding information (e.g., education, income, and occupation) was ignored. Thirdly, our sex-stratified analysis shows that O_3_ may be associated with an elevated risk of fracture in females but not in males. Further studies are required to elucidate why females may be more affected by O_3_ mechanistically, as well as its potential relationship with the higher prevalence of osteoporosis in females than in males.

In summary, our findings prove that O_3_ exposure elevates the fracture risk. This study suggests that more attention should be paid to strengthening air pollution control to prevent fracture occurrence. Our findings could also facilitate policy-making decisions toward controlling air pollution as a risk factor for bone health.

## Data availability statement

The original contributions presented in the study are included in the article/Supplementary material, further inquiries can be directed to the corresponding author.

## Ethics statement

Ethical approval was obtained from Beijing Jishuitan Hospital Ethical Review Committee (No. 202009-16). The patients/participants provided their written informed consent to participate in this study.

## Author contributions

SL, RX, and XJ conceived and supervised the study. SL and RX designed the study and wrote the manuscript. SL, RX, MG, YZ, and NL performed study. JC, XL, and XJ developed new software and performed simulation studies. SL, RX, JC, XL, and XJ analyzed data. XJ made manuscript revisions. All authors reviewed the results and approved the final version of the manuscript.
