# Supplementary material for: Risk of ozone exposure-induced fracture
Source: Front Public Health. 2023 Mar 16;11:1153256. doi: 10.3389/fpubh.2023.1153256 (PMC10061083; doi:10.3389/fpubh.2023.1153256)
Supplement: Supplementary file 1 [file Data_Sheet_1.pdf]

Supplementary materials

Table S1. ORs and 95% CIs of fractures associated with each IQR increase in the ozone exposure at different lags.

Table S2. Estimated ORs of the fracture onset associated with each IQR increase in the ozone exposure, stratified by gender.

Table S3. Estimated ORs of the fracture onset associated with each IQR increase in the ozone exposure, stratified by age.

Figure S1. The spearman correlation coefficients between different air pollutants and temperature.

Figure S2. Estimated ORs and 95% CIs of the fracture onset associated with each IQR increase in the ozone exposure, stratified by gender.

Figure S3. Estimated ORs and 95% CIs of the fracture onset associated with each IQR increase in the ozone exposure, stratified by age.

Table S1

ORs and 95% CIs of fracture onset associated with each IQR increase in the ozone exposure at different single lags.

Lag	ORs	95% CIs
Lag 0	0.96	(0.90, 1.02)
Lag 1	1.00	(0.97, 1.03)
Lag 2	1.03	(1.00, 1.06)
Lag 3	1.04	(1.01, 1.07)
Lag 4	1.03	(1.01, 1.06)
Lag 5	1.01	(0.98, 1.04)
Lag 6	0.97	(0.92, 1.02)

Table S2

Estimated ORs of the fracture onset associated with each IQR increase in the ozone exposure, stratified by gender.

Lag	Male (ORs)	Female (ORs)	*P* for heterogeneity
Lag 0	0.99	0.92	0.30
Lag 1	1.03	0.97	0.07
Lag 2	1.05*	1.01	0.12
Lag 3	1.06*	1.03	0.40
Lag 4	1.04*	1.03	0.81
Lag 5	1.00	1.02	0.52
Lag 6	0.94	0.99	0.43

* $P < 0.05$

Table S3

Estimated ORs of the fracture onset associated with each IQR increase in the ozone exposure, stratified by age.

Lag	Age ≥ 60 (ORs)	Age < 60 (ORs)	*P* for heterogeneity
lag0	0.97	0.95	0.78
lag1	1.00	1.00	0.83
lag2	1.01	1.04*	0.49
lag3	1.02	1.05*	0.55
lag4	1.03	1.04*	0.81
lag5	1.02	1.00	0.49
lag6	1.01	0.95	0.32

* $P < 0.05$

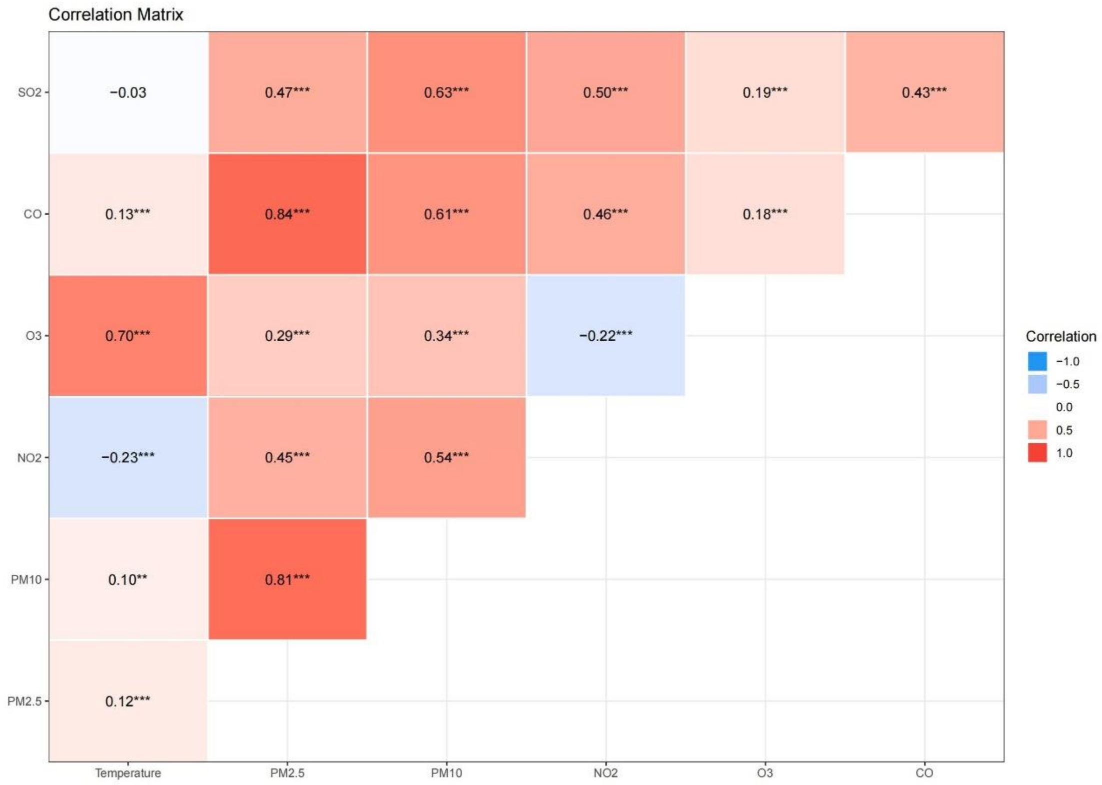

Figure S1.

Spearman coefficients of the correlations between different air pollutants and temperature.

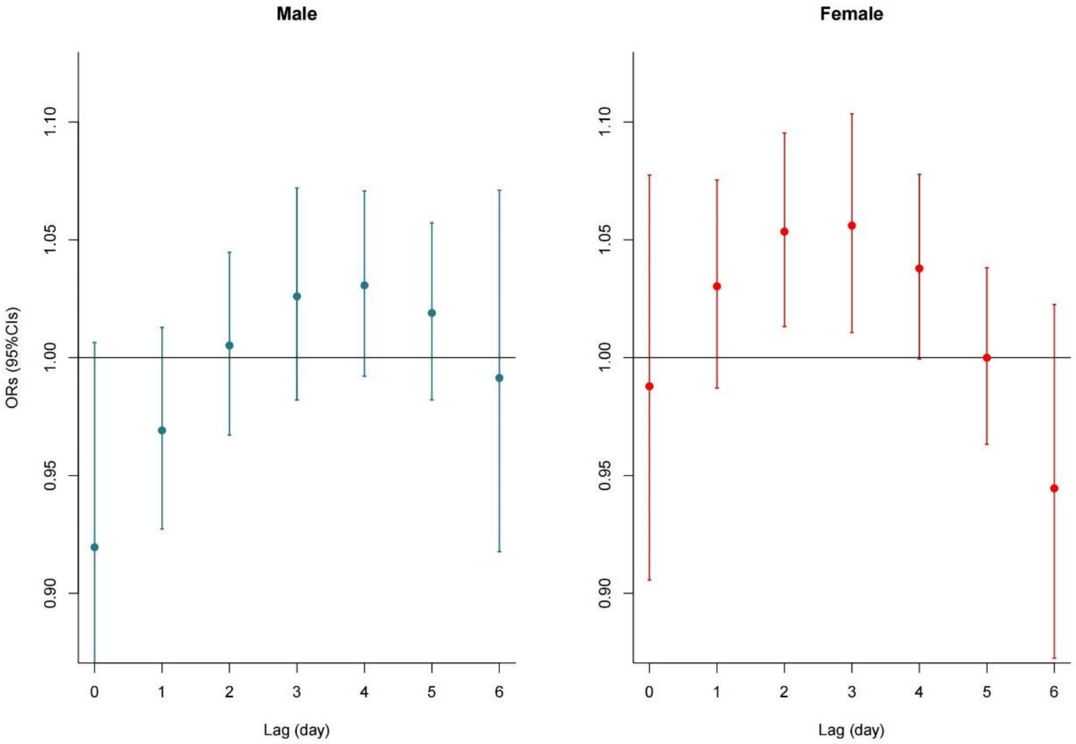

Figure S2. Estimated ORs and 95% CIs of the fracture onset associated with each IQR increase in the ozone exposure, stratified by gender.

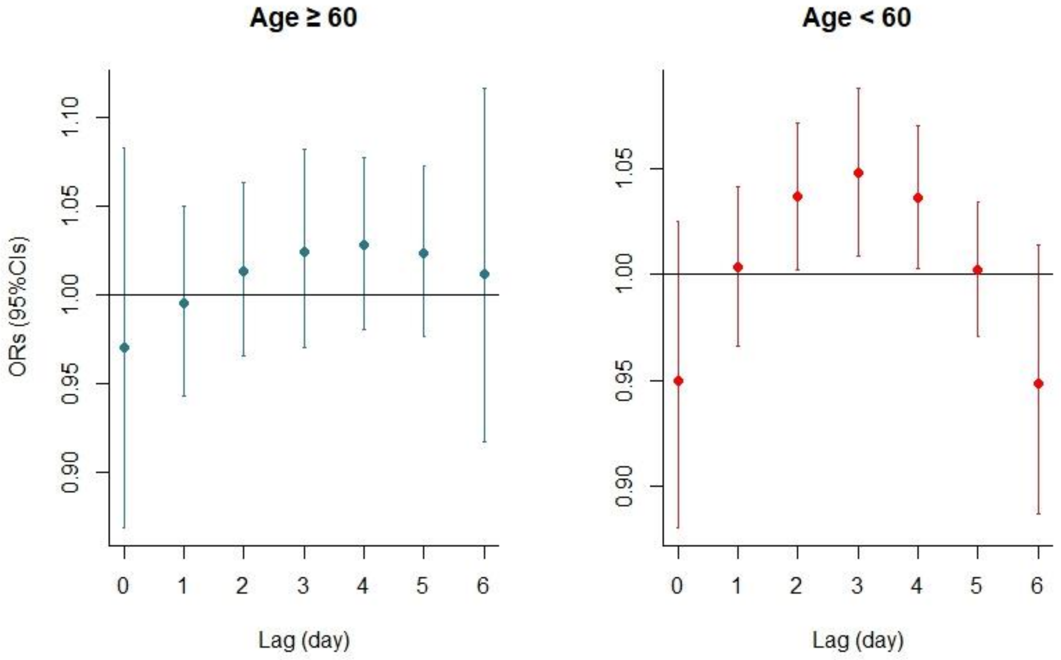

Figure S3. Estimated ORs and 95% CIs of the fracture onset associated with each IQR increase in the ozone exposure, stratified by age.
